# STAT1/3/5 Functions Driving Lipid and Energy Metabolism in Cancer and Immunity

**DOI:** 10.3390/ijms27062828

**Published:** 2026-03-20

**Authors:** Mark Rinnerthaler, Martina Sykora, Anastasios Christoforakos, Fritz Aberger, Gerardo Ferbeyre, Richard Moriggl

**Affiliations:** 1 Department of Biosciences and Medical Biology, Paris Lodron University of Salzburg, 5020 Salzburg, Austriafritz.aberger@plus.ac.at (F.A.); 2 Department of Biosciences and Medical Biology, Cancer Cluster Salzburg, Paris Lodron University of Salzburg, 5020 Salzburg, Austria; 3 Center for Tumour Biology and Immunology, Paris Lodron University of Salzburg, 5020 Salzburg, Austria; 4 Centre de Recherche du Centre Hospitalier de l’Université de Montréal (CRCHUM), Montreal, QC H2X 0A9, Canada; 5 Département de Biochimie et Médecine Moléculaire, Université de Montréal, Montreal, QC H3C 3J7, Canada

**Keywords:** metabolic syndrome, metabolic liver cancer, cancers obesity connex, metabolism and immunity, JAK-STAT1/3/5 lipidomics, Warburg effect, hydride transfer chain, Lipid droplets

## Abstract

Lipids are the major energy reservoir, but excessive fat accumulation drives immune cell trapping, chronic inflammation, autoimmunity, and cancer. Lipid synthesis, secretion, degradation, and the shuttling to cellular organelles and compartments are still poorly investigated in all cell types of the mammalian body. The major routes of FA uptake are dietary uptake, lipolysis, and de novo synthesis. We highlight disease associations zooming in on the Signal Transducer and Activator of Transcription 1/3/5 (STAT1/3/5) molecules in association with cytokine, growth factors, and hormone action, steering lipid metabolism. We compare STAT-lipid crosstalk from nuclear and mitochondrial perspectives, highlighting roles in immunity, metabolic diseases, and cancer, and providing insights into key regulatory mechanisms of lipid metabolism. A high degree of cellular flexibility in metabolic adaptation explains the need for fine-tuning, in which STAT molecules can function as rheostats to maintain energy equilibrium within cellular compartments. This concept bridges, e.g., high-energy flux or the Warburg effect, with the Hydride Transfer Complex upon low-energy provision. Another interesting STAT1/3/5 aspect is their Lipid droplet (LD) association and LD formation. LDs play key roles in disease initiation or progression, including autoimmunity or cancer, as well as chronic inflammatory diseases due to their role in (1) lipotoxicity, (2) cell death regulation, (3) immune system amelioration, and (4) energy provision. Finally, the therapeutic consequences of the angles are outlined, along with future research directions.

## 1. Introduction: Metabolism Control in Mammals and a Link to STAT1/3/5 Signalling

### 1.1. Basic Principles of Metabolism and Lipid Function

**Figure 1:**
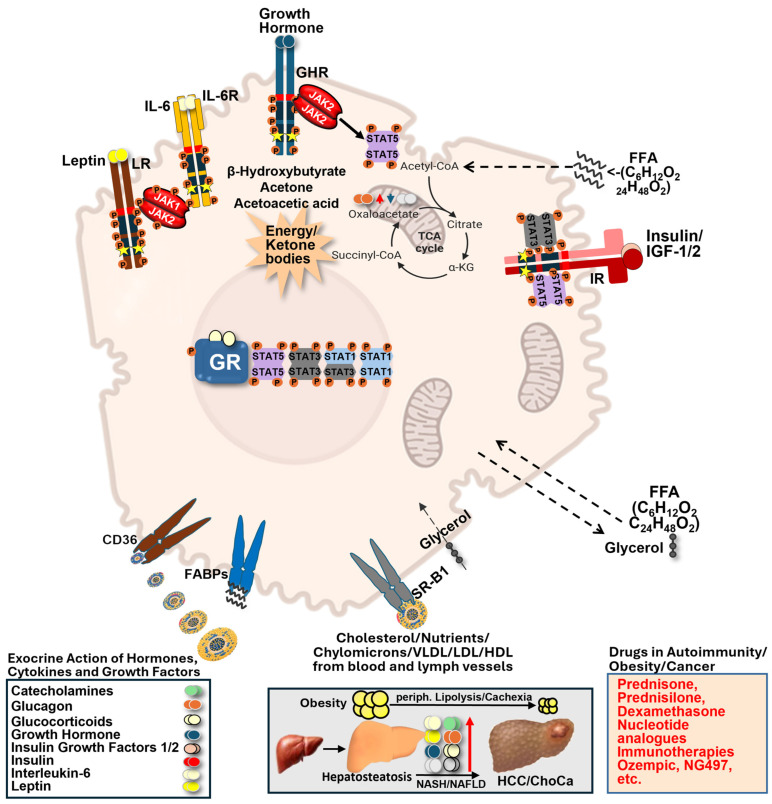
**JAK–STAT1/3/5 signalling and metabolism in white and brown adipocytes with liver epithelia.** Fine-tuning of metabolism via cytokines, growth factors, hormones, and steroids is a hepatic lipid metabolism affair. A liver crosstalk to adipose tissues is also neuro-endocrine wired, impacting back on immunity, where we depict major lipid cell types in **Figure 2**. Regulation by many signalling pathways (to simplify, we exclude, e.g., RAS-RAF-ERK or PI3K-AKT-mTOR-S6 kinase cascades in the drawing) is crucial for energy homeostasis and the prevention of metabolic diseases. Most energy within the body, and key innate immune cell components such as complement proteins, originate in the liver due to nutrient uptake (major sources: lipid particles, glucose, amino acids, etc.), as well as lipid, glycogen, and protein synthesis. Large particle lipid depots represent high-energy cargos, illustrated by chylomicron or HDL/LDL uptake via SR-B1 and CD36 surface receptors, which are also present on these particles, facilitating uptake, in addition to other lipid or free fatty acid (FFA) influx via FABPs. The liver can uptake lipids and generate energy from them. Additionally, it can both synthesize and catabolise lipids (e.g., β-oxidation and TCA cycle). However, different compartments are used for lipid anabolism or catabolism: lysosomes, mitochondria, and peroxisomes catalyze lipid degradation; the ER-GOLGI pathway is used together with the cytoplasm for lipid anabolism (some lipid anabolic reactions were also reported to occur in mitochondria, e.g., cardiolipin synthesis). Thus, opposing lipid-metabolism processes can happen in parallel and simultaneously in the liver epithelium. A specific, highly vascularized capillary system in the liver distributes metabolites and fuel within the body. The liver senses also infectious diseases and can react with inflammation, during which, e.g., the secretion of inflammatory cytokines such as IL-6, G-CSF, TNF-α, IFNs, or IL-1β follows. Excessive nutrient uptake or problems in JAK–STAT signalling (like GH resistance) can promote fatty liver disease, metabolic syndrome, and up to liver cancer development (**grey box**). A fraction of obese patients displays chronic inflammation hallmarked by NASH/NAFLD, contributing to enhanced liver cancer formation. Metabolically active liver cancer displays peripheral lipolysis and end-stage cachexia in progressed/advanced cases. Examples of drugs that impact lipid metabolism are summarized in the red box. Exocrine actions of nuclear hormone receptor ligands, GH and Leptin (these are cytokine examples), and Insulin/IGF-1/2 as growth factors are indicated in the boxed legend, with colour-coded symbols. JAK1/2 tyrosine kinase actions are simplified illustrations for major cytokines with metabolic action, indicating substrate phosphorylation by orange-circled phosphate groups. Growth factor receptors can activate STATs without JAK kinase activation through their intrinsic kinase domains. (Created partly in Biorender. Rinnerthaler, M. (2026) https://BioRender.com/51ytjnq, accessed on 14 March 2026).

**Figure 2:**
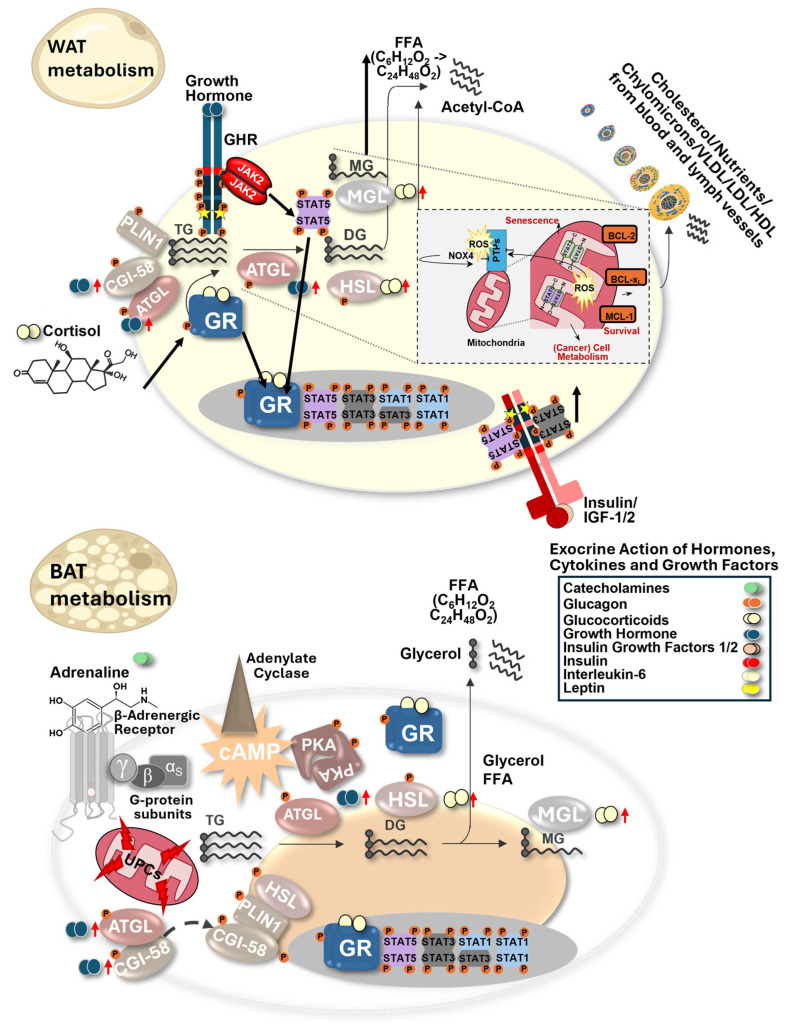
**JAK–STAT1/3/5 signalling and metabolism in white and brown adipocytes.** Key molecule names are placed into context between a white (**WAT**, upper) and brown (**BAT**, lower) adipocyte. Exocrine actions of nuclear hormone receptor ligands, GH and Leptin as cytokines, and Insulin/IGF-1/2 as growth factors, with colour-coded symbols, are indicated in the boxed legend. Interplay with stress hormones (corticosteroids and catecholamines) and their interaction with STAT TFs happens via GR binding. Major kinase phosphorylations from tyrosine and serine/threonine kinases converge on key enzymes and substrates (indicated by orange circled phosphate groups). Thus, catabolic and anabolic functions are interwoven with GTPase and kinase action converging on TFs such as the STATs or nuclear hormone receptors. This also includes ligation of hormone receptors via sex-specific hormones (not depicted) and GH action, which is much higher and more pulsatile in males than in females. GH also regulates dehydrogenases (p450 cytochrome components) and other metabolic enzymes in a sex-specific manner, with GR interplay. Within a reductionist view, we only indicate GH signalling in WAT and β-adrenergic receptor signalling as major examples in BAT with STAT5 function for thermogenesis in BAT or lipolysis in WAT via the three major lipases. Importantly, STAT molecules are not only TFs; they also possess mitochondrial functions. They can translocate to the mitochondria, which is depicted in a simplified manner for a WAT cell only. The function and membrane potential of mitochondria are regulated by BCL-2 family members that antagonize apoptosis, as outlined in WAT. Survival and proliferation are largely under the control of JAK–STAT signalling, in which AP-1 or high c-MYC TF levels in many cell types of the body, including blood cells, depend on transcriptional regulation by STAT1/3/5 molecules, which impact on the TCA cycle in mammalian cells, complicating metabolic views. (Created partly in Biorender. Rinnerthaler, M. (2026) https://BioRender.com/u78qqck, accessed on 14 March 2026).

Lipids, carbohydrates, nucleic acids, and proteins are the main biomolecules that can serve as fuel for cellular metabolism. Fat is the largest reservoir of energy, even in lean individuals, but large fat depots can lead to immune cell trapping, chronic inflammation, accelerated autoimmunity, and increased cancer rates. Major lipid types in cellular metabolism are fatty acids (FA), triacylglycerols, phospholipids, bioactive lipid messengers, and cholesterol, which are also regulated by cytokines, hormones, and growth factors. These signals promote largely cellular differentiation, proliferation, and survival via the STAT protein family, and they are also involved in differentiation or immune cell function, regulating immune exhaustion or active killing processes (**Figure 1** and **Figure 2**).

### 1.2. The JAK–STAT Signalling Pathway

The family of Signal Transducer and Activator of Transcription (STAT) proteins is a critical downstream target of cytokine and growth factor signalling pathways and is characteristically activated by Janus kinases (JAKs). Thus, upon ligand binding to its cognate receptor, the associated JAK kinases phosphorylate STAT proteins, leading to dimerization, nuclear translocation, and transcriptional activation. The classic JAK/STAT signalling pathway links external signals to transcriptional control of metabolic, survival, and immune functions. Examples include virally infected or cancer cells, where STAT family members are critically involved in Interferon/Interleukin/protein hormone or growth factor signalling, clearing abnormal cells. STAT transcription factor (TF) shuttling to mitochondria controls the respiratory chain and mitochondrial gene transcription, largely of tRNA and rRNA.

### 1.3. STAT Proteins Beyond Their Nuclear Role

Importantly, STAT proteins are not restricted to their nuclear transcription factor function but can also localize to other cellular compartments, including mitochondria, where they directly influence metabolic processes. A set of TFs can reside not only in the cytoplasm and the nucleus, but also in the mitochondria, where they can regulate metabolism in versatile ways, altering the respiratory chain or the Krebs cycle [1]. STAT1/3/5 possess cytoplasmic and mitochondrial metabolism steering and metabolic status sensing capacity, which is probably a more ancient STAT affair. The most conserved STAT protein is STAT3, which exists in single-cell amoebae, millions of years before tyrosine kinases evolved, suggesting a function independent of its parallel nuclear dimer role as a TF. STATs can enter mitochondria, and many metabolic functions are controlled therein [2,3,4], but STATs can also propagate transcription via their TF function, which shapes metabolism in mammals. These observations indicate that STAT proteins integrate transcriptional and mitochondrial regulation of metabolism.

### 1.4. Interaction with Other TFs

Key TFs that are most frequently mutated are the tumour suppressor protein Tumour Protein 53 (TP53), and MYC amplifications, MYC translocations, or other genetic changes, such as extrachromosomal MYC, leading to very high MYC (family member) oncoprotein levels in cancer. MYC family members regulate, e.g., nucleotide and glutamine metabolism, and cytokine/growth factor-activated STAT3/5, or copy number gain of STAT3/5 can boost MYC levels, which are essential for many cancer progression pathways [5]. Thus, STAT signalling does not act in isolation but interacts with major oncogenic and metabolic transcriptional networks.

### 1.5. Integration of Transcriptional and Mitochondrial STAT Functions

STAT proteins also regulate mitochondrial membrane integrity by transcriptionally upregulating BCL-2 family members (**Figure 2**, **upper**), thereby directly facilitating survival. STAT5 can also indirectly control BCL-2 family member transcription by repressing miRNA15/16, thereby impairing survival protein expression and reducing BCL-2 family member mRNA transcription [6]. Despite the involvement of multiple transcription factors, STAT proteins retain a unique role due to their ability to couple extracellular signalling directly to both transcriptional and mitochondrial metabolic regulation. In general, activation or repression of gene transcription by STAT family member TFs that are weak transactivating molecules themselves requires cell-type-specific recruitment of co-activators (p300/CBP, Cbp/p300-interacting transactivator with Glu/Asp-rich carboxy-terminal domain 2 (CITED2), etc.) or co-repressor molecules (Nuclear Receptor Corepressor 1/2 (NCOR1/2)/BCL6-Corepressor (BCOR), etc.). This is incompletely studied in disease contexts, but cell-type-specific molecular interactions exist that are DNA-locus-dependent and also dictated by epigenetic gene regulation.

### 1.6. Metabolism in Physiological and Disease Contexts

Metabolism control is a fundamental part of living matter, intrinsic to the regulatory control of a single cell. However, fat metabolism in a mammal is much more complicated, e.g., to protect the skin surface from pathogens or to wire important metabolic organs, such as the liver, stomach, colon, or pancreas, with tissue-microenvironment interactions for food digestion, nutrient uptake, and storage. These processes become particularly relevant in pathological conditions such as cancer, metabolic syndrome, and chronic inflammatory diseases, where metabolic homeostasis is disrupted. Here, one should add that muscle tissue requires a substantial amount of fat, demonstrating the need for lipid fuels coming largely from adipose tissue. Skeletal muscle tissue uses crosstalk with cytokines, adipokines, and myokines, such as Leptin, Adiponectin, and Irisin, which impact mitochondrial muscle cell activity during exercise via STAT1/3/5, AMP-activated protein kinase (AMPK), Peroxisome proliferator-activated receptor gamma coactivator 1-alpha (PGC-1α), and Sirtuin 1 (SIRT1) signalling [7]. Therefore, metabolism must be balanced, and in mammals it is regulated by kinases and TFs, as well as enzyme turnover, post-translational modification, etc. Often, cell type and disease context dependencies exist in lipid metabolism, but how it interconnects with main organs, cell types in health or disease, and ageing remains an intensive area of investigation.

Steering cellular functions such as survival, proliferation, and differentiation, and their biology, therefore requires significant control of lipid metabolism, a process that is incompletely studied and often not yet proven in molecular detail. We focus on how STAT1, STAT3, and STAT5 regulate lipid metabolism at transcriptional and organelle levels, and how these functions contribute to metabolic reprogramming in cancer and immunity.

## 2. Pathophysiology and Versatile Metabolism in Cancer Cells

### 2.1. Obesity, Metabolic Imbalance, and Cancer Risk

One can view fatty liver disease or pancreatitis as pre-neoplastic diseases with disturbed metabolism. Metabolic organ problems are associated with higher driver mutation burden, e.g., in pre-neoplastic regions of the pancreas, where Kirsten Rat Sarcoma Viral (KRAS) mutations are found to be involved in adenocarcinoma initiation and progression in aged and more obese individuals [8]. Our Western-style diet promotes high obesity rates, which is much higher in the USA, but Europe is following that trend. Obesity is accompanied by a higher risk of developing autoimmune diseases, which is higher in the USA population compared to Europe, linked with lower Body Mass Index in the European population [9]. Autoimmunity is associated with worse prognosis in several cancer types, partly because the immune system is overactivated and “trapped”. Large granular lymphocyte leukemia (LGLL) predisposition is, e.g., 4× higher in individuals associated with rheumatoid arthritis. LGLL is characterized by high *STAT3* gain-of-function mutations, which facilitate DNA hypermethylation associated with increased methionine metabolism [10]. It was demonstrated that metabolic dysregulation, immune imbalance, and oncogenic signalling pathways are closely interrelated rather than independent. For example, changes in lipid metabolism, such as those that occur during obesity, directly impact immune cell composition and tumour microenvironments. Regarding obesity, larger fat accumulations change, e.g., lymphoid (T cells) or myeloid subtypes and activity status, resulting in chronic inflammation. Here, increased T helper 1 responses and more myeloid-derived suppressor cells home to fat tissue. Examples of obesity-related cancer types are pancreas, stomach, esophagus, hepatocellular, cholangio, colorectal, breast, prostate, ovarian, or cervix carcinomas, among others. Increasing attention is necessary for pancreatic ductal adenocarcinoma (PDAC) due to its high malignancy and lack of efficient therapies, dominantly driven by KRAS oncoproteins. PDAC will climb to the 2nd most common cancer killer in the next decade, and high body mass index (BMI) is associated with higher cancer predisposition. Similarly, the frustrating increase in colorectal cancer incidence in patients under the age of 45 may be at least partly related to diet and lifestyle and resulting changes in the microbiome, posing a challenge to the healthcare system and calling for in-depth causal studies of metabolic alterations [11,12,13].

### 2.2. Organ Dysfunction and Lipid Overload in Metabolic Disease

Beyond systemic effects, metabolic overload also directly alters organ function, particularly in the liver as a central hub of lipid metabolism. Part of the tumour-promoting effect of fat, metabolic syndrome, and obesity also lies in the higher energy flux that facilitates neoplasia. Long-term overnutrition results in massive lipid depots that serve as reservoirs for immune cell trapping. Moreover, cells with very high fat content, e.g., hepatocytes in fatty livers, increase in volume drastically (**Figure 1**). This creates problems with liver epithelial signalling due to increased cell sequestration. In addition, liver epithelium is loaded with large fat droplets, best seen on histologic sections, in fat-loaded hepatocytes with micro- and macrovesicular steatosis in non-alcoholic fatty liver disease (NAFLD). Sections display lipid-loaded compartments with large hydrophobic masses and areas. However, all biochemical reactions require aqueous solutions, and the liver epithelium cannot perform key reactions important for whole-body metabolism. Thus, inappropriate liver function upon high fat content can have drastic life-shortening consequences, where in the USA, kids with NAFLD display a shorter life expectancy than their parents, since the liver runs into metabolic problems up to metabolic liver cancer progression. The NAFLD hepatocytes cannot detoxify the body effectively, e.g., via the urea cycle and p450 dehydrogenases, as key hepatic processes are impaired or continually damaged by high fat masses, which disrupt vital hepatic function. The liver may also fail to produce sufficient vitamins or sex hormones, and it may impair immunity due to problems in complement protein and amino acid synthesis, negatively impacting lymphocyte populations. Furthermore, blood clotting factor synthesis can be impaired in fatty liver conditions.

### 2.3. Metabolic Plasticity in Cancer and Immune Cells

Similar metabolic adaptations are also required in immune cells, which must dynamically reprogram their metabolism in response to activation state and the microenvironment. The situation in immune cells, e.g., T cells, is similar. A resting or a fully activated T cell needs to change its metabolism to shape activity against cancer cells. These processes are incompletely understood in ageing organisms, where immunity, physical activity, and tissue regeneration decline, all under the control of GH and stress hormone action. To make things worse, ageing is exacerbated by the increased prevalence of metabolic disease. Type-2-diabetes (T2D), obesity, autoimmunity, neurodegeneration, or cancer onset increase with age. The extent of mitochondrial or DNA damage that accumulates with ageing is individual, and its contribution to disease onset is poorly understood. Importantly, higher mutation rates happen in rapidly dividing cell types due to stem cell turnover. Surface epithelial cells accumulate driver mutations over time, which ultimately converge on key cancer pathways, forming carcinomas [14,15]. Therefore, more knowledge of epithelial metabolism would be key. Obesity, T2D, and metabolic syndrome (**Figure 1**) can facilitate liver cancer initiation and progression, providing a significant health risk [16]. Identifying and targeting aberrant metabolic processes is difficult because metabolism is conserved in normal and diseased cells, as well as in immune cells. Unwanted toxicity or immunosuppression can result from imbalanced metabolism, which needs to be avoided. Treatment options exist to interfere with metabolism to target the excessive demand for nutrients in some cancer types (**Figure 1**). The success of anti-obesity therapy lately might tell us in decades if BMI normalization by, e.g., incretin mimetics or incretin enhancers, was beneficial to fight obesity-associated cancer types. Newer developments also focus on key lipid enzyme inhibitors, e.g., Adipocyte Triglyceride Lipase (ATGL), which catalyze triglyceride breakdown to LDs targeted by NG497, a current ATGL inhibitor explored in clinical testing [17]. Moreover, corticosteroids are powerful, effective drugs with pleiotropic metabolic effects, often combined in chemotherapy regimens and frequently selected for their immunosuppressive action. They can help to block chronic inflammation-driven cancers, e.g., ameliorating problems associated with infectious pathogens. At later disease stages, metabolic dysregulation manifests systemically, for example, in cancer-associated cachexia. Here, peripheral lipolysis and muscle waste are prominent (**Figure 1** and **Figure 2**). White (WAT) and brown (BAT) adipose tissue are derived from a mesenchymal stem cell, which can also give rise to muscle cells. This suggests a similar origin and greater metabolic similarity in fat metabolism within the mesenchymal lineage. However, we focus only on lipolytic activity in WAT and BAT to better explain lipolysis in major lipid depots in mammals (ignoring muscle tissue, which also serves as a metabolic reservoir in aggressive cancer; **Figure 2**). Moreover, the amount, location, and inflammatory status of fat tissue itself are crucial for disease outcomes. Taken together, these examples highlight the remarkable metabolic flexibility of cancer cells and their surrounding microenvironment. Cancer cells are highly flexible, switching their metabolic wiring upon need. A cancer cell that undergoes rapid or slow proliferation, that has an excess or a lack of nutrient or oxygen supply, or that undergoes senescence while in a dormant/quiescent stage, has a different metabolic wiring. Thus, within the same cancer cell type, changes in metabolism impact back on the microenvironment or it has immune cell consequences, as exemplified by lactate production in hypoxic conditions, which promotes immunosuppression. Cancer cells, together with immune cells, might be the best example of how their versatile proliferation and survival change across different immune or cancer biology processes.

### 2.4. Lipid Metabolism and Metastatic Progression

The metabolic flexibility described above not only supports tumour cell survival but also enables key processes such as metastatic dissemination. Metastasis is the most dominant clinical problem in cancer patients, and it often requires a metabolic switch, such as a shift toward fat metabolism, to enable cell migration and adaptation to distinct environments. It is also noteworthy that cancer cells depend on dietary fatty acids to initiate metastasis. Specifically, palmitic acid, a major component of palm oil, can enhance the metastatic potential of these cells. Meanwhile, inhibition of the fatty acid receptor, the “Cluster of Differentiation 36 receptor” (CD36), on cancer cells completely prevented metastasis in mouse models of oral, skin, and breast cancer. Mechanistically, CD36 facilitates the uptake of fatty acids by these metastatic cells, which are then metabolized by beta-oxidation to provide the substantial energy required for the inefficient process of metastasis [18,19].

### 2.5. Metabolic Reprogramming: The Warburg Effect

This dynamic metabolic reprogramming is classically captured by the Warburg effect, which describes a shift from oxidative phosphorylation to glycolysis even in the presence of oxygen. Biological processes such as metastatic dissemination, immune cell escape, or tumour tissue invasion in nutrient-rich or nutrient-poor environments require metabolic adaptation. This was recognized early and is known as the “Warburg effect” [6,20]. The Warburg theory of cancer has long been widely criticized, and it took >70 years (through transgenic mouse model work) to prove that Otto Warburg was largely right. The Warburg effect underscores the strong evolutionary conservation of metabolic regulation, but its consequences for cells undergoing glycolysis can be diverse, and it remains poorly understood in mammals and in disease. Yeast, as a simplified eukaryotic model, has a related process known as the Crabtree effect, discovered by Herbert Grace Crabtree. In both mammalian and yeast cell systems, cells shift their energy metabolism from mitochondrial oxidative phosphorylation (OXPHOS) toward glycolysis, producing lactate/ethanol. Certain metabolites, such as lactate, serve today as biomarkers and can provide a readout of the dynamic phenotype of immunosuppression. Well-studied examples of immunosuppressive agents include lactate, adenosine, kynurenine, and cholesterol [21,22]. However, whereas yeast cells actively inhibit mitochondrial respiration, mammalian cells maintain functional mitochondria, with respiration being downregulated. This is reflected in 2–4 mol ATP instead of 32 mol ATP produced by glycolysis, leading to increased lactate production. One can also view ATP production as versatile in mammalian cells embedded within tissues, and certain conditions require a kind of rheostat metabolic control.

## 3. Glycolysis and Lipid Anabolism in Cytoplasm via the Hydride Transfer Complex Reaction

### 3.1. From Glycolysis to Anabolic Metabolism

**Figure 3:**
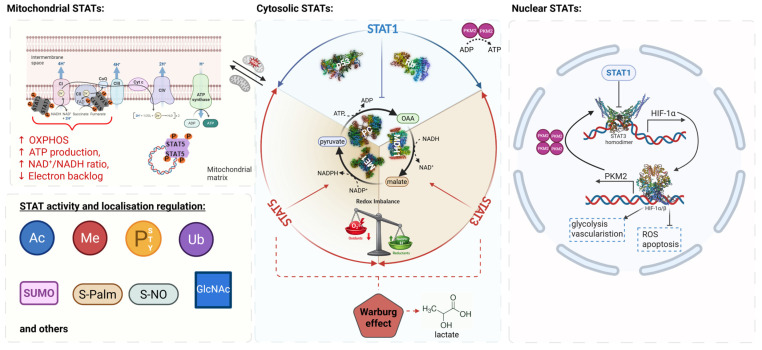
**STAT1/3/5 interplay with energy provision via mitochondria, the Hydride Transfer Complex, or by Warburg cycle reactions.** Normal or cancer cells can generate large amounts of energy-rich molecules, such as ATP, NADH, or GTP, for energetic processes when mitochondria are intact, and nutrient/metabolite flux is high, usually under high blood vascularisation and O2 availability. This facilitates high metabolic rates, rapid proliferation, and enhanced survival. However, when mitochondria are impaired/damaged, or during senescence, cells can switch to slower forms of metabolism, such as the Warburg cycle or the Hydride Transfer Complex (HTC) reaction The HTC converts NADH into NADPH, representing lipid anabolism in the cytoplasm. This results in lower energy production, and we suggest, speculatively, that the HTC can be induced by oncoproteins, such as high STAT3/5 activity/expression on chromosome 17q, which is frequently gained in cancer. STAT3 is paradoxically a tumour suppressor upon PTEN loss in prostate cancer (PCa) [25], and this can also lead to high HTC activity upon damaged mitochondria. HTC activity can be repressed by the tumour suppressors TP53 (pdb: 1TUP), located on chromosome short arm #17p, frequently lost or mutated in cancer, retinoblastom protein (RB) (pdb: 4ELL), or possibly STAT1 (the latter not investigated, but also frequently locus methylated or lost in cancer). The HTC is a holoenzyme protein complex key for lipid anabolism: Tertiary structures of both Malate Dehydrogenase 1 (MDH1; 334 amino acids; pdb: 7RM9) and Malic Enzyme 1 (ME1; 572 amino acids; pdb: 7X11) reconstitute dimers, and tertiary structure of Pyruvate Carboxylase (PC; 1178 amino acids; pdb: 8XL9) forms a tetramer in one HTC holoenzyme. As a result, transfers of hydrides (H^−^) from NADH to NADP^+^ originate. Thus, the HTC consumes NADH and liberates NADPH, restoring redox balance. In parallel, the HTC fuels antioxidant systems with their essential cofactor, NADPH. Sensing by TP53 or RB results in transcriptional repression of the three enzymes that form the HTC, limiting NADPH levels to counteract ROS-dependent damage and promoting oncogene-induced senescence. Cancer cells lacking TP53 or RB display high HTC activity post-translationally and can efficiently convert NADH into NADPH, maintaining redox balance and thereby neutralizing ROS-mediated DNA damage, phosphatase inactivation, and lipid peroxidation. Subsequently, cancer cells can undergo senescence despite mitochondrial dysfunction, continuing to proliferate and survive at a slower rate than with ATP, distinct from the Warburg effect. Thus, the HTC reaction may facilitate selection pressure, e.g., during metastatic dissemination or under drug-induced selection, warranting further work. Thus, the context and activity of driver mutations, as well as the expression levels of STAT1/3/5, are important factors in determining whether oncogene-driven cells undergo senescence or progress through the cell cycle. The classic concept of STATs being at receptors only and then going to the nucleus is outdated, and one could picture TP53 regulating STAT protein turnover, localization, and activity through PTMs. The STAT molecules are about twice the size of TP53 and are heavily modified by PTMs, as indicated by public Mass Spectrometry data. They remain poorly investigated and adopt a dimeric or tetrameric structure that binds to chromatin similarly to TP53 or NFATs. We use three examples of versatile intracellular metabolic steering functions of STAT1/3/5: (**I**) Most sensor systems for low nutrient supply, e.g., O-GlcNACylation, are found on STAT1/3/5A/5B and STAT6 [26,27]. Thus, O-GlcNACylation of serine or threonine residues on many proteins (e.g., STATs) can act as a nutrient sensor, particularly sensitive to ambient glucose. (**II**) Another nutrient-sensing system is STAT3 expression/activation levels in PCa cells [25]. Loss of Stat3 in a Pten^−/−^ mouse promotes PCa due to reduced LKB1/pAMPK with simultaneous activation of mTOR/CREB. Energy stress promotes AMPK activation, which phosphorylates SREBP-1, Acetyl-CoA Carboxylase (ACC), and ATGL. ACC is a multifunctional holoenzyme complex catalyzing carboxylation of Acetyl-CoA to malonyl-CoA upon energy production stress as a rate-limiting step in fatty acid (FA) synthesis [28]. This results in PCa metastasis, whereas persistent pY-Stat3 elevates LKB1/pAMPK levels and blocks the mTORC1/CREB pathway, preventing PCa development. (**III**) A third regulatory function of STAT3/5 was described, involving oncogenic and inflammatory signals that dominantly induce nuclear pY-STAT3/5 (pdb: 6QHD), driving HIF-1α/β expression [29]. HIF-1α/β (pdb: 9OFU) homo- and heterodimers promote increased glycolysis and neo-vascularisation. HIF-1 is regulated by O_2_ presence and connected to NFE2-Like BZIP Transcription Factor 2 (NRF2), essential for Redox balance. This can initiate a positive feedback loop between HIF-1, subsequently driving the expression and activation of Pyruvate Kinase M2 (PKM2) and STAT3. Subsequently, PKM2 can associate with persistent levels of pY-STAT3 in the nucleus, acting as a dual kinase, and has been described to phosphorylate Histone H3 at Thr11, altering the epigenome [29,30]. Cytoplasmic PKM2 functions as a pyruvate kinase transferring a phosphoryl group from phosphoenolpyruvate (PEP) to ADP. This generates ATP, but when PKM2 forms a tetramer, it has high ATP activity. STAT5A/B activity can drive HIF-1 expression similarly to pY-STAT3, but activated STAT1 represses HIF1A, and high STAT1 expression antagonizes STAT3 due to heterodimerisation. Last, but not least, the balancer function of STAT5A over the more dominant oncoprotein STAT5B is regulated by wild-type TP53 and by SOCS1, which is frequently methylated or lost in cancer. In summary, high levels of ROS and NADH induce senescence in a TP53- and RB-dependent manner, and their metabolism is under STAT1/3/5 control downstream of cytokine, growth factor, and hormone action. Thus, metabolic control is essential and versatile in normal, cancer, and immune cells. (Created in Biorender. Rinnerthaler, M. (2026) https://BioRender.com/d3ho67v, accessed on 14 March 2026).

**Figure 4:**
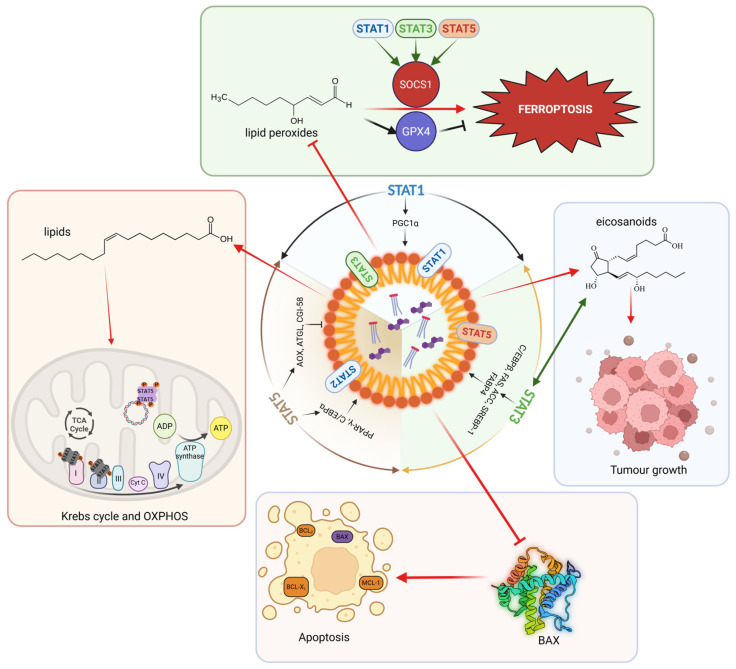
**Lipid droplets synergize with STAT1/3/5 pathway activation and mitochondrial activity.** LDs are organelles that besides lipid storage have versatile functions such as blockage of ferroptosis by acting as a buffer for polyunsaturated FA, modulation of apoptosis (e.g., redirecting the pro-apoptotic protein BAX (pdb: 1F16) from mitochondria to LDs), feeding lipids into mitochondria providing Acetyl-CoA essential also for epigenetic gene regulation and open chromatin formation, building eicosanoids that promote tumour growth in concerted action with STAT3 action that can also promote eicosanoid production (**green arrow**) or STAT3/5 both driving OXPHOS together requiring mitochondrial STAT3/5 function (**red arrow**). Both SOCS1 and STAT1 are tumour suppressor proteins that antagonize and negatively feed back on oncogenic STAT3/5 signalling. Interestingly, SOCS1 and GPX4 are also involved in ferroptosis and/or survival processes, and SOCS1 is transcriptionally regulated by STAT1/3/5. Key gene products transcribed by STAT1/3/5 are shortlisted, as introduced in **Figure 1** and **Figure 2** and in the text’s body. LDs can also take up ROS-damaged lipid peroxides, e.g., as seen in NASH/NAFLD, metabolic syndrome, HCC context, or in WAT tissue of obese individuals, thereby scavenging reactive lipid species and maintaining redox balance. Biochemical experiments provide evidence that STAT1/2/3 proteins are present on the lipid monolayer surface of LDs, most likely due to the versatile roles of STAT5A/B in lipid metabolism. We postulate that STAT5 may also be present on the outer wall of LDs, though this remains to be experimentally verified. (Created in Biorender. Rinnerthaler, M. (2026) https://BioRender.com/4zv38ps, accessed on 14 March 2026).

Although the Warburg effect refers to the shift in metabolism toward glycolysis with lactate production, it does not elucidate the mechanisms by which metabolic intermediates are diverted to meet anabolic demands, such as lipid synthesis. For example, cancer or immune cells must meet energy demands with biosynthetic demands. Therefore, rather than rehashing the shift to glycolysis, it is vital to recognize the mechanisms by which glycolysis-derived metabolic intermediates are diverted to meet biosynthetic demands or to regulate cellular redox status. The Warburg and Crabtree effects, as explained in different models, share a fundamentally similar mechanism of glycolysis [23,24]. Immune cells or cancer cells can gain an advantage by switching to lactate metabolism. We understand today that they can change metabolic fates, membrane synthesis/fortification, neo-vascularization, etc., upon hypoxic conditions. Cancer cells also evade immune cell recognition by counterattacking via lactate secretion as an immunosuppressive metabolite. The STAT transcription factors are key regulators of metabolic plasticity in both cancer and immune cells. STAT TFs are like rheostats, since their distinct phosphorylations and other post-translational modifications alter their activity (**Figure 3** and **Figure 4**).

Moreover, their translocation to various cellular compartments, including most organelles and the nucleus, is possible. However, they may also reside in the cytoplasm under regulatory control by kinases, GTPases, and metabolic enzymes that attach post-translational modifications. Fat metabolism is a slower but more persistent and dense form of energy provision, due to the smallest volume of stored fuel. Energy is mainly generated in cells through mitochondrial activity (apart from peroxisomal β-oxidation or lysosomal lipid/protein degradation), but if mitochondria are damaged or malfunctional, then cytoplasmic lipid metabolism plays a key role in helping cancer cells survive a shortage of mitochondrial metabolite flux. Interestingly, lipid anabolism can still occur in the cytoplasm and endoplasmic reticulum, but lipid catabolism primarily occurs in mitochondria, peroxisomes, and lysosomes. Here, lysosomes are used for receptor-mediated endocytosis at acidic pH, which is mediated by Lysosomal Acid Lipase (LAL), which degrades lipids. Lysosomal lipid degradation or β-oxidation ultimately produces Acetyl-CoA. This can also serve as a starting point for new lipid synthesis, but, when needed, Acetyl-CoA can be produced from monosaccharides via glycolysis or from amino acids, again highlighting the TCA cycle’s role.

Thus, lipid metabolism might be more flexible than, e.g., amino acid or carbohydrate metabolism, which depend more on mitochondria or on uptake from the microenvironment/bloodstream. However, the spatial organization of lipid anabolism or catabolism requires independent regulation, as YIN/YANG pathways rely on sensor proteins to complete the necessary energy cycles. STAT molecules can also orchestrate energy availability within cells and can slow down or speed up energy supply sensing metabolism via post-translational modification, expression, activity, and cellular organelle or compartment localisation (**Figure 3**).

### 3.2. The Hydride Transfer Complex as a Metabolic Hub

In situations of mitochondrial dysfunction, such as hypoxia or defects in oxidative phosphorylation, cells must compensate for metabolic shortages by rearranging cytoplasmic metabolic pathways. One of these compensatory mechanisms, described by the Gerardo Ferbeyre lab, is the establishment of a new metabolic complex, the Hydride Transfer Complex (HTC; **Figure 3**), which facilitates lipid anabolism while maintaining redox homeostasis. Mitochondrial dysfunction can develop due to hypoxia/too high reactive oxygen species (ROS) production, or loss of mitochondrial STAT3, which facilitates HTC formation (**Figure 3**) [31]. The HTC supports lipid anabolism via three enzymatic reactions that catalyze a metabolic cycle that transfers the hydride ion (H^−^) from NADH to NADP+. While the NAD+ generated by HTC supports the Warburg effect, based on increased glycolysis, it can also support lactate assimilation, which has recently been recognized as an important metabolic trait in many cancers. Mechanistically, HTC links NAD+/NADH and NADP+/NADPH metabolism, thereby coupling glycolytic flux to reductive biosynthesis and antioxidant defence. Tertiary structures of HTC are formed both by Malate Dehydrogenase 1 (MDH1) and Malic Enzyme 1 (ME1) as reconstituted dimers, and the tertiary structure of Pyruvate Carboxylase (PC) forms a tetramer complex within one HTC. Thus, the HTC reaction is steered in a holoenzyme complex, with two dimeric and one tetrameric assembly forming possibly a ring-like structure for catalysis (**Figure 3**). Expression of these three enzymes matters, and whether they are expressed at normal levels or upregulated at the protein level needs to be determined. Overall, this is underexplored in the context of cancer or immune cells. Nowadays, we often measure cellular states indirectly via RNA transcripts, but the HTC reaction or STAT1/3/5 activity and localisation are quantitative protein consequenes that require consideration of post-translational mechanisms.

### 3.3. STAT-Dependent Regulation and Open Mechanistic Questions

It is currently unclear how STAT3 or possibly STAT5 influence the expression and assembly of HTC [31], but several non-mutually exclusive mechanisms can be envisioned. These include transcriptional regulation of HTC component enzymes, modulation of post-translational modifications that affect enzyme assembly, and indirect effects through changes in cellular redox state and metabolite availability. However, direct experimental evidence linking STAT activity to HTC formation or function remains limited. It was also described, mainly in cancer cell line studies, that steering of energy metabolism via STAT1/3/5 molecules occurs partly through specific and distinct post-translational mechanisms and subcellular localisation, which can transport STAT family members to different cellular compartments (**Figure 3**). Here, phosphorylation and O-GlcNACylation, as well as lipidation, acetylation, ubiquitylation, and methylation, or different translational initiation with alternative methionine *START* codons, were described, as were C-terminal splicing products that lack, e.g., the critical mitochondrial Ser727 within STAT3β, affecting cancer or immune cell consequences distinctly [32,33]. Alternative explanations, including ATPase impairment and lower ATP levels, can account for sensing via metabolic STAT3 action. STAT3 enhances lipolysis in mature adipocytes by increasing ATGL protein expression, while simultaneously inhibiting Tumour Necrosis Factor α (TNF-α), which also increases inflammation-induced lipolysis [34,35]. In pre-adipocytes, the function of STAT3 is rather clear as C/AAAT Enhancer Binding Protein β (C/EBPβ) is a direct target gene of STAT3, which is activated, e.g., by Interleukin (IL)-6. STATs in general can induce several C/EBP family members in different cell types or cancers, which subsequently switch on metabolic gene regulatory programs. In the case of C/EBPβ induction by STAT3, it promotes adipogenesis during adipocyte differentiation, most likely via concerted action with adipogenic Peroxisome Proliferator-Activated Receptor Gamma 2 (PPAR-γ2) induction [36]. Taken together, the HTC represents a potential metabolic node that integrates glycolysis, redox control, and lipid biosynthesis, positioning STAT proteins as context-dependent modulators. When metabolic needs shift from ATP to NADPH production in cancer or immune cells, metabolic plasticity becomes crucial for adapting to environmental pressures such as hypoxia or nutrient stress. The HTC can thus be considered an adaptation in cellular metabolism that alters energy management by coupling ATP consumption with NADPH production. The adaptation enables the assimilation of glucose and lactate while also increasing lipid anabolism and ROS defence capability.

## 4. Extracellular Signals Steering Metabolism

### 4.1. Hormonal and Cytokine Control of Metabolism

Cytokines and growth factors act in concert, often accompanied by steroid/stress hormone interplays converging on TF interplays (**Figure 1**). Sex hormones, metabolic hormones such as tetraiodothyronine (T3/T4) secreted by the thyroid gland, and stress hormones, rapidly released from the adrenal gland and under control of the hypothalamus–pituitary–adrenal (HPA) axis, display neuroendocrine wiring. These signalling systems do not act in isolation but converge on shared intracellular pathways, with STAT proteins representing key mediators linking extracellular cues to metabolic gene regulation. Metabolic hormones and their nuclear hormone receptors can also bind to kinases, thereby regulating metabolism, e.g., Thyroid Hormone Receptor binding to Pyruvate Kinase M2 (PKM2; **Figure 3**) or GR binding to LCK tyrosine kinase at the T Cell Receptor, thereby blocking its activity without ligand stimulation by stress hormone. Anterior pituitary gland secretory cytokines, such as Prolactin and Growth Hormone (GH), have key metabolic regulatory functions and neuroendocrine crosstalk with stress hormone release. Both Prolactin and GH activate STAT5 (also STAT1/3), which can interact with nuclear hormone receptors via their N-terminal domains. The latter are activated by steroid ligands, explaining synergies in metabolic reactions. A well-known catabolic function is, e.g., the synergistic action of Insulin with GH in the breakdown of lipids [37]. These examples highlight that STAT activation occurs within complex hormonal environments, where combinatorial signalling determines metabolic outcomes in a context-dependent manner. Many hormones are made from cholesterol precursors (e.g., corticosteroids, progesterone, 17-beta-estradiol, testosterone, 9-cis retinoic acid, triiodothyronine, thyroxine, etc.). Overall, the interplay between nuclear hormone receptors and STAT signalling in cytokine, growth factor, and hormone contexts is poorly studied in a sex-specific manner, regulating key processes, such as drug metabolism, redox balance, and detoxification, as well as general metabolism.

### 4.2. Integration of Signalling Pathways and STAT Activation

Central signalling pathways exist, including Rat Sarcoma (RAS)–Rapidly Accelerated Fibrosarcoma (RAF)–Extracellular Signal-Regulated Kinase (ERK), Phosphoinositide 3-Kinase (PI3K)–AKT Serine/Threonine Kinases (AKT)–mTOR (mechanistic Target Of Rapamycin)–S6-Kinase, and JAK–STAT1/3/5 axis [2,3,4]. Rather than acting independently, these pathways form highly interconnected signalling networks that converge on transcriptional regulators, including STAT proteins. Triggering of these pathways results in the activation of many kinases and GTPases (e.g., Kinases: MAPKs, ERK1/2, JNK1/2/3, p38A-G, JAK1/2/3/TYK2, AKT1/2/3, PI3K, RAF-family kinases; GTPases: RAS-, RHO/RAC/CDC42-, RAN-RAL-GTPases, etc.), where ATP and GTP are required for signalling capacity. Signal integration boils down to distinct sets of TFs that are typically activated by these enzymes and GTPase function. The latter are usually also required for proper traffic and compartment localization within the cell. This is well illustrated for nuclear import and export by the GTPases Rat Sarcoma (RAS)-related Nuclear proteins (RAN) or RAS-Like proteins (RAL). STAT proteins, through their SRC Homology 2 domain (SH2, binding phosphorylated tyrosines) and rapid activation dynamics, serve as key amplifiers of tyrosine kinase signalling and integrate signals from multiple upstream pathways. There are also important functions of Activator Protein 1 (AP-1, composed of JUN/FOS family members, also under STAT1/3/5 transcriptional control) or NFκB family members, which can cause, e.g., higher proliferation, migration, or chronic inflammation. Interaction with these TFs can occur with STAT1/3/5 in either antagonistic or synergistic transcriptional modes, changing inflammatory fates, and this is dependent on the target gene locus, the cell type, and the specific protein composition within transcriptional complexes.

### 4.3. Metabolic Consequences of Signal Integration

Metabolome analysis tells us what is happening in the biological systems being studied. Cancer cell-immune cell interactions are often embedded in an immunosuppressive environment. Extracellular signalling ultimately translates into metabolic phenotypes that define cellular behaviour in both cancer and immune cells. Here, particularly mesenchymal lineages such as vasculature or fibrotic tissue, impact the metabolism of tumour tissue. Adipocytes are also mesenchymal and more polymorphic in the disease/cancer context. One could simply look at their size and lipid species/proteome content, which differ vastly. Cancer-associated fibroblasts can execute immunosuppressive functions [38]. However, whether adipocyte differentiation and function also significantly impact immunity in specific cancers remains understudied and is quite likely due to lipid depot locations in frequent sex-specific cancers or in metabolic liver cancer in obesity, where high lipid metabolism occurs. This highlights a critical gap in our understanding of how extracellular signals shape lipid metabolism in the tumour microenvironment.

Metabolome provides the closest link to the phenotype resulting from the interaction between the genome and the environment. Metabolites themselves can influence metabolic processes, whether DNA, RNA, or proteins are synthesized or broken down, as explained, e.g., by methionine metabolism, which provides methyl groups for DNA or RNA methylation, steering the epigenome. The Human Metabolome Database states that the human body contains 253,245 metabolites (HMDB, 17.03.2026, https://hmdb.ca/statistics). Targeting metabolic pathways has been applied across different fields of life sciences. Biomarker discovery, tracking drug effects, identifying targets for drug development, and serving as prognostic parameters are among the ongoing applications in metabolism research, where lipid metabolism is most versatile [39].

## 5. Metabolic Immune Cell Consequences

### 5.1. Metabolic Requirements of Immune Cell Function

Immune cells have the task of protecting and detecting pathogens, and they reside within the hepatic vasculature, where many immune cells patrol. Moreover, immune cells need to eliminate tumour or infected cells. They need to be flexible since they travel, for example, from the lymphoid organ, into the vasculature, and from a kind of resting state to the malignant tissue site. This functional plasticity requires dynamic metabolic reprogramming to support migration, proliferation, and effector functions. Oxygen or pH changes affect their metabolism. In particular, Natural Killer (NK), Cytotoxic T Lymphocyte (CTL), and γδ T cells require activation and direct contact with the target cell to execute killing. Here, γδ T cells possess individual, organ-specific functions to recognize, e.g., phospholipid species as commensal antigens, mounting immunity. Macrophages have greater phagocytic activity, but lipid catabolism will be required if, e.g., bacteria or virus-hosting cells are engulfed.

### 5.2. Metabolic Constraints in the Tumour Microenvironment

Despite their intrinsic flexibility, immune cells often encounter severe metabolic constraints within the tumour microenvironment (TME). Immune cells often do not reach the target, and cancer cells are embedded in a rich extracellular matrix that must be degraded by immune cells before they can encounter the cancer cells, all of which are energy-demanding processes. Thus, cancer cells shield themselves from immune attack by a collagen-rich protein matrix. Immune cells need to “chew off” the matrix via protease secretion, and they uptake small peptides from the matrix via SLC transporters, which can fuel the TCA cycle during energy demand, as described for proline cycle loops as the main amino acid component, apart from glycine, in collagen fibres [40]. Immune cell dysfunction and metabolic problems in immune cells can converge and manifest clinically. Inefficient immune-cancer cell recognition and killing of cancer cells, or immune cell exhaustion or inactivation by metabolites, display immune challenges. These distinct cellular states can lead to an immunosuppressive TME. The success of immunotherapy has demonstrated that cancer can be cured when immune cells efficiently kill cancer cells. Therefore, targeting not only the metabolic pathways in cancer cells but also those in the TME, and understanding the metabolic needs of immune cells to rescue immune failure by supplying missing metabolites are today’s avenues in cancer research. These also entered GMP manufacturing processes for engineered immune cells, particularly in light of essential amino acid requirements [41], but lipid requirements for activating or blocking immunity are less well understood.

### 5.3. Systemic Influences: Infection, Ageing and Metabolism

Beyond the local tumour environment, systemic factors such as infection, nutrition, and ageing further shape immune cell metabolism. Many diseases can have immune cell type imbalances, e.g., in obesity, autoimmunity, and chronic inflammation during ageing. Ageing is significantly accompanied by clonal haematopoiesis, and immune cells can become exhausted or malfunction. At the cellular level, these alterations are reflected in defects in immune cell differentiation and function. For example, incomplete antibody chain production observed in multiple myeloma reflects impaired B cell maturation and metabolic dysregulation, often preceded by clonal expansion within the hematopoietic system. The metabolic consequences of viral or bacterial infection are also well known and can be illustrated by the saying “Feed a cold, starve a fever,” which refers to glucose or fat metabolism benefits or disadvantages, depending on the pathogen and disease status. Mouse model work revealed distinct wiring for bacterial or viral pathogens, a poorly understood phenomenon. Here, a more rapid clearance of virally infected cells is highly glucose-dependent, whereas bacterial infections depend more on ketogenesis [42]. This illustrates that nutrition is important in disease. Most therapies do not take this into account, and they are influenced in cancer patients with additional autoimmune disease or in aged individuals who could suffer from other complications. Chronic inflammation or chronic infectious disease status due to microbes or viruses will complicate interference strategies. In the end, an individual who cannot cope with metabolic control is doomed to disease. Neurodegeneration, infectious disease, autoimmunity, cancer, and chronic inflammatory diseases are all facing distinct metabolic states aggravated by ageing. The most versatile cells to rapidly adapt to harsh milieus are aggressive, advanced cancer cells or activated immune cells. Both need to penetrate tissues to clear an infection or survive it. Prominent microbe examples include *Helicobacter pylori*, *Fusobacterium nucleatum* (Fn), and/or chronic *Epstein–Barr virus* (EBV) infection. These are particularly associated with gastrointestinal (GI) cancer progression or, in the case of EBV, also lymphomagenesis. EBV is also linked to autoimmunity. In this context, it is interesting to note that many immune cells are GI tract-associated. Immune repertoires are in Peyer’s patches, gut-associated lymphoid tissue, or within the epithelia and GI tract mucosa. The GI tract is essential for nutrient uptake, and bile acids enter the lymphatics and transport the lipids within the biliary epithelial cell system into the liver, where bile acids are recycled. Infectious disease is also, to a large part, a JAK–STAT affair. This is well reflected in Interferon signalling, where STAT1/2/3 and IRF9 transcribe signals from the many Type 1 Interferons and from Type 2 Interferon Gamma, the latter largely depends on STAT1, helping to clear infection through their transcriptional and metabolic programmes. Also, STAT4/5/6 are involved in the regulation of Interferon synthesis. The metabolic consequences of cytokine action are studied mainly in single-stimulation experiments. However, the interactions of Leptin, IL-6, Interferons, GH, Prolactin, and STAT with nuclear hormone receptors, and the synergistic or antagonistic actions with growth factors, leave us with much more experimental work ahead to illuminate them. It is tricky for cancer or immune cells to survive harsh environments, and despite necrosis, pH, O2, and nutrient gradients, metabolic demands create selection pressure for the outgrowth of the fittest cells. Importantly, cancer stem cells can undergo dormancy or senescence in the absence of nutrients, propagating a silent state that is difficult to kill with drugs such as chemotherapeutic agents. Thus, placing patient-specific immune cells into experiments with more complex stimuli for metabolism steering is key to investigating, e.g., in normal or cancer organoid work, leaving room for discoveries.

## 6. Mitochondrial STAT Action and Metabolic Crosstalk with the JAK–STAT Pathway

STAT proteins are not restricted to their canonical nuclear transcription factor roles but can also localize to mitochondria, where they directly influence metabolic processes, particularly the respiratory chain and the Krebs cycle. These observations indicate that STAT proteins integrate transcriptional and mitochondrial layers of metabolic regulation, thereby linking extracellular signalling to intracellular energy homeostasis.

### STAT-Specific Mitochondrial Mechanisms

STAT1: Hepatic mitochondria from STAT1-deficient mice show both defects in oxidative phosphorylation and increased numbers of mitochondria, resulting in higher PGC1α levels, also due to direct transcriptional STAT1 induction by Interferons [43]. PGC1α is a mitochondrial coactivator that also binds PPAR-γ and the cAMP Responsive Element Binding Protein 1 (CREB), a TF activated by Protein Kinase A (PKA) in BAT (**Figure 2**, **lower**), thereby steering mitochondrial biogenesis. STAT1 behaves as a tumour suppressor in most cancer types, except in NK cells, where STAT1 loss leads to a response to MHC class I-negative cells [44]. Thus, STAT1 activation promotes upregulation of MHC class I, which is essential for foreign antigen presentation upon infection or neoantigen recognition in cancer. Thus, pY-STAT1 (phosphorylated tyrosine) transcribes Major Histocompatibility Complex (MHC) class I components, which are suppressed by increased pY-STAT3 activity, as shown, e.g., in Tasmanian devil schwannoma transmissible cancer growth [45], since STAT1 forms a heterodimer with STAT3, altering also tyrosine kinase inhibitor resistance [46]. At the metabolic level, STAT1 displays context-dependent functions that extend beyond classical immune regulation. STAT1 was reported to induce adipogenesis in mature adipocytes by inhibiting lipolysis upon IFN-γ stimulation. Here, pY-STAT1 binds to the promoter region of PPAR-γ2, which is an activator of adipogenesis. STAT1 also binds to the promoter region of Lipoprotein Lipase (LPL), a rate-limiting factor in triglyceride hydrolysis [47]. On the contrary, during differentiation of pre-adipocytes, STAT1 is barely expressed, but Interferon (IFN)-γ-induced pY-STAT1 can still suppress adipogenesis during maturation [48].

STAT3: STAT3 was intensively studied for mitochondrial impact, and it is associated with respiratory complex chain I and II protein–protein interactions regulating OXPHOS and mitochondrial protein translation (**Figure 3** and **Figure 4**), but it does not directly regulate mitochondrial genes as TF [49]. Thus, in contrast to its nuclear role, mitochondrial STAT3 primarily functions through protein–protein interactions rather than direct transcriptional control. Mitochondria divide separately from normal cell division, and they can be amplified in response to increased activity demand, including high mitochondrial mass in cancer types, to support efficient lipid anabolism. Importantly, RAS-RAF oncoprotein transformation depends on mitochondrial STAT3 activity [50]. Therefore, STAT3 is quite different for mitochondrial wiring compared to STAT5 proteins [51], which were shown to function directly as TF in mitochondria. STAT3 might move into mitochondria as an anti-parallel dimer upon Ser727 phosphorylation, which is essential for mitochondrial function, but Ser727 phosphorylation may not be required for transport, as newer findings suggest [49]. Similarly, STAT1 shares the conserved Ser727 site, but it is less studied in mitochondrial function. Both STAT1 and STAT3 are phosphorylated on Ser727 by stress, DNA damage-induced, or metabolic kinases.

STAT5: STAT5 is involved in mitochondrial function, but the exact mechanisms and the specific effects of STAT5A/B in different cell types are poorly understood. In contrast to STAT3, STAT5 was shown to transcribe mitochondrial tRNA and respiratory chain genes. We lack understanding of how STAT5 traffics to the mitochondria and whether it requires specific post-translational modifications, but pY-STAT5 has been shown to interact with the Dihydrolipoyl Acetyltransferase (PDC-E2) in mitochondria. PDC-E2 normally resides in the mitochondrial matrix, where it converts pyruvate to Acetyl-CoA, but it can also go nuclear, where it has been shown to interact with STAT5A in mature adipocytes, participating in gene transcription. This dual localization of PDC-E2 further illustrates the tight coupling between mitochondrial metabolism and transcriptional regulation [52,53]. Mitochondrial STAT5-mediated transcription or repression might require parallel dimerization and coactivator (e.g., PGC1α) or corepressor recruitment, or interaction with other mitochondrial transcription factors, to efficiently alter mitochondrial RNAs due to poor STAT5A/B transactivation domains. However, it is not known whether mitoSTAT5 is differentially spliced or processed, how it is modified in detail to regulate these processes, or what the more oncogenic STAT5B does, which is dominant in liver metabolism. In summary, PDC-E2–pY-STAT5 interaction was described to promote a metabolic shift from oxidative phosphorylation to glycolysis, which represents another metabolic steering by a STAT member.

In summary, the roles of mitochondrial STAT action need to be seen in a context-dependent manner. For instance, STAT1 is generally known to play a tumour-suppressive and immune-activating role, whereas STAT3 and STAT5 are generally known to play pro-survival and anabolic metabolic roles. However, these roles are not fixed and can vary based on cellular context, metabolic status, and microenvironmental cues. Therefore, mitochondrial STAT signalling can be considered a flexible system that adjusts metabolic and transcriptional outputs in response to environmental and cellular demands.

## 7. Molecular Wiring with STAT1/3/5 Family Members Steering Lipid Metabolism

### 7.1. Hormonal/Cytokine Control of Lipid Metabolism via STAT Signalling

The interaction between immune cells or cancer types and hepatic architecture is key to providing fuel to cancer or immune cells. GH is an important modulator of hepatic energy metabolism, acting in a pulsatile, sex-specific manner in males and females. This endocrine regulation is tightly linked to STAT5 activation, positioning STAT proteins as central mediators of hormone- and cytokine-driven metabolic control. This occurs in all mammals, with strong, high-pulse rates in males and more continuous, lower-pulse rates in females. Both STAT5A and STAT5B are sex-specific TFs, and hepatic STAT5B is much more highly expressed than STAT5A; the latter is dominant in mammary gland development and is missing in marsupials that lack mammary glands. This explains the unique STAT5B-driven liver epithelium. Surprisingly, the liver vasculature is also special, displaying a specific dominance of STAT5B repressing, e.g., G-CSF, essential for neutrophil generation and activation, in combination with CD14-ligated lipopolysaccharide exposed on Gram-negative bacteria upon infection [54]. GH action alone is anabolic, e.g., for tissue regeneration/muscle building, but it acts catabolic in combination with Insulin for long-chain lipid breakdown. Here, corticosteroid action can also synergize with both to facilitate more efficient and rapid lipid mobilization from fat stores, where STAT5 interacts with the Glucocorticoid Receptor (GR) (**Figure 2**). These observations demonstrate that STAT signalling integrates multiple hormonal inputs to fine-tune lipid metabolism in response to physiological demand.

### 7.2. Liver-Specific STAT Functions in Metabolic Regulation

STAT-dependent metabolic regulation is highly tissue-specific, reflecting differences in organ function and cellular composition. The liver represents a central organ for lipid metabolism, where STAT5B plays a dominant regulatory role. Cre recombinase-mediated double deletion of STAT5A/B specifically in hepatocytes led to elevated hepatic de novo lipogenesis by inducing PPAR-γ [55]. Increased de novo lipogenesis was accompanied by steatosis, T2D, and hyperlipidemia at older ages. Independently, we observed that hepatic deletion of STAT5 led to defects in bile acid homeostasis, reduced serum triglyceride (TG) levels, and dysfunctional enterohepatic bile acid recycling [56]. Animals deficient for the GHR (**Figure 1** and **Figure 2**), a major upstream activator of STAT3 and STAT5A/B, exhibited a similar phenotype [57]. STAT5B was shown to be indispensable for controlling hepatic functions. Disruption of hepatic GH–STAT5–IGF-1 signalling leads to GH resistance, which is a whole-body condition, since GH acts on all organs and cell types except neurons [56]. GH resistance is, e.g., characterized by hepatic failure of GH signalling, leading to postnatal dwarfism. GH levels are neuroendocrine signals of GH resistance, and more GH is produced, which remains functional in conditional transgenic mice or GH-resistant patients, leading, e.g., to peripheral lipid mobilization. Impaired GH signalling or heterozygosity of IGF-1 is associated with short stature, prolonged lifespan, reduced sex hormone synthesis (associated with infertility), and other consequences, including effects on immunity/infectious diseases. GH resistance is clinically relevant, as tight correlations with the onset of metabolic syndrome have been observed [58]. The transcriptional up- or downregulation of GH-STAT5 signalling by genetic variants in STAT5B has been demonstrated in human population studies [59]. Collectively, we observed in this patient study the association between variants in the STAT5B/STAT5A/STAT3 locus on the long arm of chromosome #17 and changes in TC, low-density lipoproteins (LDL-C), high-density lipoproteins (HDL-C), and triglyceride TG levels in five European populations with replication in the Global Lipids Genetics Consortium data.

Furthermore, a sixth independent cohort was included, revealing that a STAT5B SNP (rs8082391) was associated with reduced serum TC and LDL-C levels. Similarly, we were able to show, using a sensitized phenotypic mouse screening approach for gene dosage at the STAT3/5 locus on mouse chromosome #11, that STAT5 copy number variations are associated with immune cell hypersensitivity in allergic reactions and with metabolic syndrome induction upon a high-fat diet [60,61]. We showed that copy number gain in #17q, where both STAT3 and STAT5 are located, is frequent in lymphoid cancers [62,63,64], but the lipid-metabolism consequences were not investigated.

In summary, genetic association studies link STAT5 expression particularly to cholesterol biosynthesis, impacting hepatocytes and biliary epithelial cells involved in bile synthesis. Similarly, reports indicate that a defective GH–GHR–JAK2–STAT5B axis can impair the Hepatocyte Nuclear Factors (HNF)-Small Heterodimer Partner (SHP)-Liver X Receptor (LXR) TF network, eventually leading to altered serum lipid levels. Interestingly, the LXR–Retinoid X Receptor (RXR) interaction as well as Carbohydrate Response Element-Binding Protein (ChREBP) promote both lipogenesis and bile acid formation, whereas Farnesoid X Receptor (FXR)-RXR interaction causes bile acid efflux with accompanying diminished gluconeogenesis, bile acid and cholesterol synthesis, and ablated lipogenesis [65,66]. Lipid storage, glucose and FAA uptake, as well as Insulin sensitivity, are, on the other hand, regulated by the interaction between PPAR-γ and RXR. Hepatic GH signalling also upregulates ribosomal biogenesis and sexually dimorphic gene expression, promoting male- or female-specific gene regulation. Here, e.g., major proteins in urine or p450 drug-metabolizing enzymes/dehydrogenases are sex-specifically driven by GH pulses, resulting in sex-specific drug metabolism. Moreover, GH drives somatic growth by regulating proteins such as the bioactive IGF-1/-2 complexed with the Acid Labile Subunit (ALS), all of which are transcriptionally induced by hepatic STAT5B.

Furthermore, IGF-binding proteins can also be under STAT5 control [67,68]. However, the absence or reduced gene dosage of STAT5, e.g., monoallelic murine Stat5a/b deletion, also strongly impacts GH signalling on STAT1 and STAT3 activation. Here, heterozygous STAT5 expression liberates GHR binding sites [69], leading to a “false” GH gene regulation via strong STAT1/3 action, which promotes an Interferon transcriptional response. Normally, hepatic GH signalling is a dominant JAK2–STAT5B affair, which explains why STAT1/3/5 expression levels matter for cytokine or growth factor responses. GH-STAT5 signaling can promote repression of the Sterol Regulatory Element Binding Protein 1c (SREBP-1c), thereby blocking de novo lipogenesis. Many nuclear hormone receptors can interact with STAT5 via the N-terminal tetramerization domain. Furthermore, STAT1 and STAT3 also interact with nuclear hormone receptors, thereby steering fat metabolism. Transcriptional interaction outcomes are also dictated by the recruitment of corepressors or coactivators, which are essential for gene and epigenetic regulation. A key metabolic control interaction that acts in synergy with STAT5 signalling is the GR. Similar hepatic conditional knockouts of the GR or STAT5 genes showed an essential interaction between STAT5 and GR for hepatic gene regulation, and such mice display a T2D phenotype [67,68].

### 7.3. Adipocyte-Specific STAT Functions in Metabolic Regulation

Both GR and STAT5 action can control peripheral lipolysis to a large extent. Various tumour models emphasised that cell-type-specific routes exist, and studies on STAT5 lipid catabolism and anabolism were published in WAT or BAT (**Figure 2**). Lipolysis in WAT and BAT is also under the control of adrenergic/cAMP-triggered Protein Kinase A (PKA) signalling that activates CREB TF. PKA activity is needed to promote Perilipin dissociation from major fat droplets, also through cofactor Comparative Gene Identification-58 (CGI-58) phosphorylation, which then docks to ATGL, the rate-limiting lipid breakdown enzyme in beta-oxidation, to promote lipid catabolism to make Acetyl-CoA. Here, ATGL can be transcribed via GR/STAT5 interaction, and PPAR-γ and C/EBPα are STAT5–GR target genes [70,71]. Both GH and corticosteroids promote efficient lipolysis in mammalian cells, acting in many tissues. It was shown that hepatic Stat5 or Jak2 loss, independent of hyperactivated GH signalling, enhances PPAR-γ expression, inducing the transcription of genes involved in fatty acid (FA) uptake. Hepatic ablation of GHR, STAT5, or JAK2 results in increased TC accumulation and liver steatosis in mouse models [57,68,72,73]. Enhanced expression of PPAR-γ and the fatty acid transporter CD36 drives fatty liver diseases. Mechanistically, CD36 is repressed by STAT5 binding to regulatory elements, and lipogenesis is catalyzed by Fatty Acid Synthase (FAS) and Stearoyl-CoA Desaturase (SCD1). Direct interaction of STAT5 with PPARG regulatory promoter regions was reported to be associated with an activating transcriptional function. STAT5 also enhances lipid metabolism, e.g., driving the transcription of Acyl-CoA Oxidase (ACOX), and represses the transcription of FAS, thereby blocking adipogenesis [74,75]. Cancer cells consume a high rate of lipids for cellular division and high growth rates, as well as for the assembly of membranes, flavin adenine dinucleotide hydroquinone (FADH2), NADPH, and Acetyl-CoA as building blocks [5]. CD36 is pY-STAT3 and pY-STAT5 regulated and an important lipid transporter that interacts with the cytoplasmic Fatty Acid Binding Protein (FABP) family members that act as lipid chaperones essential for mitochondrial function. CD36 also binds to the Scavenger Receptor class B type I (SR-BI; **Figure 1**), a high-density lipoprotein (HDL) receptor in malignant melanoma cells, to regulate cholesterol homeostasis and epithelial-to-mesenchymal transition, where both STAT3/5 are also important for promoting invasion/metastasis [76,77]. A role for IL-17-induced STAT3–FABP4 upregulation, in association with increased cellular lipid uptake, was also demonstrated [78]. To antagonize WAT or fatty liver development, BAT conversion studies and pharmacologic tools have gained increased attention, as we discuss next.

## 8. Brown Adipose Tissue Regulation by STAT Family Members

### 8.1. Functional Role of Brown Adipose Tissue in Metabolism

BAT is beneficial for mammalian metabolism and specialized for thermoregulation, with a lower fat storage capacity. BAT is used for heat generation during cold exposure.

BAT cells are very similar to muscle cells and do not store large fat depots. Regarding thermoregulation, both STAT5 and STAT6 were demonstrated to affect non-shivering thermogenesis upon deficiency of either STAT5 or STAT6 in adipocytes [71,79]. STAT5 is a key protein for acute cold-induced temperature maintenance and the induction of lipid mobilization in BAT following β3-adrenergic stimulation. We showed that mitochondrial respiration in primary, differentiated brown adipocytes lacking STAT5 was diminished. This was accompanied by increased sensitivity to cold stress upon STAT5 deficiency, associated with reduced expression of thermogenic markers, including Uncoupling Protein 1 (UCP1), while decreased stimulated lipolysis was linked to decreased PKA activity (**Figure 2**, **lower**). Brown remodelling of WAT was diminished following chronic β3-adrenergic stimulation, which was accompanied by a decrease in mitochondrial function [71]. This specialization allows BAT to rapidly convert energy into heat through non-shivering thermogenesis, making it a central regulator of systemic energy balance.

### 8.2. Integration of Immune and Cytokine Signalling in BAT Metabolism

Beyond adrenergic and metabolic signalling, BAT function is further modulated by immune-related cytokine pathways. STAT5 also plays a role in Insulin and IGF-1 signal transduction, as outlined above. This is most likely linked to IL-4/IL-13 signalling, since IRS-2 scaffold protein function is shared among these receptor systems. STAT5 is key for IL-4 signalling in T cells, steering IL-4-induced proliferation and survival, as we published in primary murine T cells [80]. Here, an interplay between STAT5 and the IRS-2 scaffold protein is possible but has not been investigated. Irrespective, IL-4 signalling contributes to thermoregulation in mammals. IL-4 can also use the IL-13Rα chain, and both IL-4 and IL-13 have been shown to exert metabolic effects on body temperature, hepatic metabolism, peripheral nutrient supply, Insulin, and glucose tolerance [81]. Additionally, upon deletion of tyrosine kinase in murine brown adipocytes, obesity results, which can be reversed by activating the Tyrosine Kinase 2 (TYK2)-STAT3 axis through constitutive STAT3 expression [82]. Importantly, the metabolic effects of STAT signalling in brown adipose tissue are highly context-dependent and may vary with environmental stimuli, such as cold exposure, hormonal status, and systemic metabolic conditions.

## 9. Lipid Droplets and ROS Metabolism, Ferroptosis and Apoptosis

### 9.1. Lipid Droplets as Regulators of Cellular Lipid Homeostasis

Another interesting aspect of lipid metabolism is LD accumulation (**Figure 4**). This is one of the lipid metabolism features in various tumours. LDs are dynamic organelles that store neutral lipids such as triglycerides and cholesterol esters, serving as reservoirs for energy and membrane biosynthesis. In cancer and immunity, LDs serve as adaptive hubs that buffer lipid overload, coordinate lipid trafficking, and modulate stress responses, thereby linking lipid metabolism to signalling and cell fate decisions. LDs are formed by synthesizing neutral lipids (both sterol esters and triacylglycerols) in the ER and transferring them to the bilayer, leading to the formation of lens-like structures. The growth of these “lenses” is supported by proteins such as Seipin, leading to LD budding. Due to this formation process, LDs are surrounded by only a single monolayer and are filled exclusively with neutral lipids. In mammals, all members of the PAT protein family (Perilipin 1–4) can be considered as LD marker proteins [83,84]. While the LD lifecycle begins in the ER, it always ends in the lysosome, where LDs are taken up by a macroautophagic process and degraded by lipases and proteases to release fatty acids for energy production [85]. Interestingly, this organelle is quite mobile and can come into physical contact with other organelles, such as the ER, peroxisomes, lysosomes, and mitochondria, via specific anchor proteins on its coat [86]. The main function of LDs is certainly their role as fat stores. They serve as storage for FA as triacylglycerols, which are released to the mitochondria for beta-oxidation when needed, providing fuel for ATP formation via oxidative phosphorylation. Furthermore, LDs naturally have a major influence on cellular fatty acid trafficking. In recent years, LDs have come into focus, with important LD functions assigned. LDs play key roles in disease initiation or progression, including autoimmunity and cancer, as well as chronic inflammatory diseases, as reflected by the following. (1) LDs function as lipid buffer: Lipotoxicity can be reduced by absorbing, e.g., lipid peroxides via LDs. (2) LDs serve as a hub for proteins with an influence on gene expression and cell death/cell survival, as well as antibacterial defence upon chronic infection. (3) Synthesis sites for the signalling lipid eicosanoid are, thus, an essential element of the immune system [83,87]. (4) LDs also serve as an energy and building block supply to fuel the Krebs cycle [86].

STAT1, STAT3, and STAT5 change the expression of genes involved in lipid metabolism, and they regulate the LD lifecycle (**Figure 4**): (i) STAT1 upregulates the expression of Cholesterol 25-Hydroxylase, LPL, and PGC1α, which are important in mitochondrial biogenesis [33,43,88]. (ii) STAT3 upregulates the expression of C/EBPβ, FAS, Acetyl-CoA Carboxylase (ACC), the master TF of lipid biosynthesis SREBP-1, the central enzymes of fatty acid beta-oxidation in mitochondria Carnitine Palmitoyltransferase (CPT1A/B), the fatty acid translocator CD36, the Fatty Acid-Binding Protein 4 (FABP4), the guanine nucleotide exchange factor VAV3, and the glucose transporter GLUT4, linking glucose and lipid metabolism [78,89,90,91]. (iii) STAT5 upregulates the glucose transporter GLUT1, and it is, in general, important for glucose or amino acid metabolism, also indirectly via MYC upregulation. STAT5 is important for the expression of key TFs such as PPAR-γ, C/EBPα/β/γ/δ, AP-1, SREBP-1, and the Acyl-CoA-Oxidase (AOX), the LD-localized lipase ATGL, and its coactivator CGI-58 [70,74,92,93]. PPAR-γ, C/EBPα/β, SREBP-1, FAS, ACC, and FABP4 have all been shown to be involved in LD biosynthesis [94,95,96,97,98,99]. Of all STAT-regulated proteins involved in lipid metabolism, the lipases ATGL with its coactivator CGI-58 and LPL play dominant roles in LD catabolism, but also AOX and PGC1α/β are essential for LD breakdown, stimulating peroxisomal and mitochondrial β-oxidation [100,101,102,103,104]. Thus, STAT5 has an indirect role in LD biology, but whether it is directly related to LDs remains to be investigated.

However, it is not only possible that the STAT family modulates the life cycle of LDs, but LDs themselves could influence the activity or trafficking of STATs. STAT1, STAT2, and Ser727-phosphorylated STAT3 have all been found on the surface of LDs [105,106]. Previously, it was shown that LDs bind other TFs such as Nuclear Factor of Activated T cells 5 (NFAT5), thereby preventing it from entering the cell nucleus [107]. Interestingly, NFAT, TP53, and STAT family members adopt remarkably similar 3D structures when bound to DNA. TP53 and STAT1/5 form tetramers when highly activated on DNA, and they also form higher-order complexes with NFAT5 in mitochondria. It is unclear whether and how that 3D protein conservation might translate into metabolism. NFAT5 is activated under hypertonic conditions and regulates the cellular stress response, triggering stress kinase activity that acts back on STAT1/3 Ser727 phosphorylation, possibly triggering mitochondrial function. By binding NFAT5, the LDs directly influence the cell’s response to osmotic stress. A future direction would be to evaluate whether LDs can function like a sponge for the STAT family, thereby influencing the STAT expression and activity profiles of cellular organelles, such as mitochondria and the nucleus. LDs regulate gene expression not only through the sequestration of NFAT5, but also through the binding of histones [108] and the release of fats that serve as ligands for PPAR-γ [109]. Finally, the STATs are epigenetic regulators as well, communicating with the histone/chromatin landscape, and LD interactions might be an important component of epigenetic gene regulation, where acetyl groups might originate.

There are many reasons why LDs accumulate particularly strongly in tumour cells. One reason is the high energy requirements of tumour cells, especially in nutrient-poor/hypoxic areas. In such cases, LDs serve as an energy reservoir by providing lipids for beta-oxidation and thus serve as fuel for oxidative phosphorylation [110]. In this context, it is also worth noting that rapidly dividing and metastasizing tumour cells require large amounts of membrane lipids to support cell division and to fortify membranes under harsh conditions that also resist immune cell attack. The FAs that are required for the formation of phospholipids are provided to tumour cells via triacylglycerols stored in LDs [111]. In the tumour (cancer cells and TME), large amounts of eicosanoids (e.g., Prostaglandin E2) are produced, as these regulate cell migration, proliferation, and immunological processes [112,113]. The precursor lipid for eicosanoid production is arachidonic acid, an important second messenger, which is released from triglycerides and thus LDs [114]. To successfully metastasize and release enough eicosanoids, the tumour is effectively forced to maintain a high number of LDs (**Figure 4**). Thus, LDs modulate the immune response and promote tumour/immune escape [111]. However, it is not only lipids that are found in LDs; the enzymes themselves (e.g., Cyclooxygenase-2 (COX2) and Prostaglandin E (PGE) Synthase), which are involved in eicosanoid production, have also been found within LDs [115]. Since LDs strongly influence lipid trafficking in the cell as a storage site for fats, they also indirectly influence other bioactive lipids, such as phosphatidylinositol and lysophosphatidic acid, affecting signalling pathways such as PI3K/AKT-mTOR and WNT signalling, which play important roles in tumour development [116]. LDs also increase drug resistance and tumour cell survival. Taken together, LDs represent more than passive fat depots: they integrate lipid storage with inflammatory mediator production, signal transduction, and therapy resistance—features that become particularly relevant under metabolic stress.

### 9.2. STAT-Regulated ROS Metabolism and Redox Imbalance

STAT1/3/5 proteins can steer anti-oxidative or oxidative processes via ROS generation both in the mitochondria and by cytokine-regulated processes in the cytoplasm, triggering, e.g., mRNA and protein expression of the NADPH Oxidase 4 (NOX4) via pY-STAT5 action [117]. NOX4 belongs to the seven members of the NOX family (NOX1–5 and DUOX1–2) that differ in cell type-specific expression and their regulatory mechanisms, where several NOX members are linked with STAT3/5 action. ROS levels affect cell-cycle progression, survival, and, in general, cytokine, growth factor, and hormone signalling. Scavenging systems such as Glutathione S-Transferase (GST) and Catalase balance intracellular ROS levels in normal cells, but excessive ROS is observed in cancer or immune cell contexts under hyperactivation, often accompanied by a deficiency in antioxidant defences. High ROS causes DNA damage, promotes mutations, oxidizes lipids, or silences phosphatases by catalytic cysteine oxidation [118,119,120,121]. Specifically, JAK2 decreases GST in epithelial cells, enhancing oxidative damage, whereas anti-oxidative scavenger function is under tumour suppressor control, e.g., hepatic JAK2–STAT5B signalling [72,122]. This illustrates that hepatic GH steers STAT5 and JAK2, which are YIN-YANG in ROS regulation, and that it is a key component in accelerating NASH/NAFLD (**Figure 2**), metabolic syndrome, and the initiation and progression of metabolic liver cancer. This redox layer provides a direct mechanistic link to LD biology, because ROS preferentially attack polyunsaturated fatty acids, generating reactive lipid species that must be buffered or removed to preserve membrane integrity.

### 9.3. Ferroptosis: Lipid Peroxidation, Iron, and Lipid Droplets as Buffers

One consequence of increased ROS production, in combination with free iron, is the oxidative degradation of lipids (especially polyunsaturated FA), leading to lipid peroxidation [123]. A regulated cell death mode called ferroptosis is activated [124] when these primarily membrane-localized lipid peroxides accumulate. By sequestering excess polyunsaturated FA and storing them in the form of neutral lipids (triacylglycerols), LDs reduce the rate of lipid peroxidation in tumour cells to suppress ferroptosis [125]. Ferroptosis is also under control via the JAK–STAT pathway, and STAT1/3/5 can induce the E3 Ubiquitin Ligase “Suppressor Of Cytokine Signalling 1” (SOCS1) [126], thereby blocking the Nuclear Factor kappa B (NFκB) inflammatory pathway. Suppressing SOCS1 via miR-210-3p promotes obesity induced adipose tissue inflammation and Insulin resistance [127]. SOCS1 is also a senescence marker/driver and is frequently lost as a negative regulator in cancer cells, either by locus methylation or genetic deletion. It can repress SLC7A11 expression, limiting cystine uptake and glutathione synthesis, the latter of which is required for the anti-ferroptosis enzyme GPX4 [128,129]. Thus, LDs and STAT-controlled redox regulators converge on a central vulnerability: the availability of oxidizable PUFA substrates, the capacity to detoxify lipid peroxides, and the integrity of cystine–glutathione–GPX4 defence.

### 9.4. Apoptosis Surveillance by LDs

Apoptosis eliminates cells with DNA damage, oncogene activation, or chromosomal defects. Blocking apoptosis is a hallmark of cancer [41]. We showed that LDs can inhibit apoptosis by removing BH3 family members (especially BAX) from the outer mitochondrial membrane and incorporating them into their own membranes. In this way, mitochondria fail to release Cytochrome C, and the formation of the apoptosome is blocked, promoting tumour survival [130]. It cannot be ruled out that the strong LD accumulation is an indirect effect in tumour cells. In many tumours, both SREBP-1 and mTOR kinase act as central sensors of nutrient perception in cells, and SREBP-1 is strongly phosphorylated/activated by mTOR under high nutrient supply. Both activated signalling pathways contribute significantly to the formation of LDs via FAS and Diacylglycerol Acyltransferase (DGAT) enzymes [111]. Adipocytes are lipid-filled cells that can release FAAs via lipolysis, which are taken up by neighbouring tumour cells and are ultimately stored as neutral lipids. This also leads to the sharp increase in LDs observed in tumour cells [131].

STAT1/3/5 are interconnected with LDs in tumour development: It was shown with triacsin C—an inhibitor of LD production—that proliferation, migration, and invasion of pancreatic cancer cells were diminished and STAT5 was linked with it [132]. Inhibition of LD accumulation induced by triacsin C or silencing of Perilipin 2 (which marks and coats LDs) sensitized cells to the chemotherapeutic gemcitabine. Interestingly, STAT5B activated the transcription of Lysophosphatidylcholine Acyltransferase 2 (LPCAT2), which drives LD production supporting pancreatic cancer chemoresistance and cell motility [132]. Together, these observations suggest two complementary layers of LD-mediated apoptosis control: (i) an acute, organelle-proximal mechanism through sequestration of pro-apoptotic factors (e.g., Bcl-2-Associated X protein (BAX)) from mitochondria, and (ii) a chronic, nutrient-driven increase in LD biogenesis via mTOR–SREBP1–FAS/DGAT signalling and lipid supply from neighbouring adipocytes.

In conclusion, LDs can be placed at the nexus of (i) lipid signalling and eicosanoid synthesis, (ii) STAT-mediated redox stress and lipid peroxidation, and (iii) regulated cell death pathways such as ferroptosis and apoptosis. Such an integrated perspective offers a mechanistic explanation for the observed accumulation of LDs in tumours and inflamed tissues, as well as how STAT pathways could modulate LD-mediated survival versus death choices in a context-dependent manner.

## 10. Conclusions and Future Directions

The interface of lipid metabolism, mitochondrial function, and cellular signalling is an important level of metabolic regulation in both tumour cells and immune cells. In this review article, we highlight the key roles of STAT1, STAT3, and STAT5 in lipid metabolism, linking extracellular signals to transcriptional control and organellar/cytoplasmic metabolic functions. A recurring theme across the discussed systems is the context-dependent nature of STAT function. STAT1/3/5 proteins can function in lipid metabolism independently of JAK kinases, playing a pivotal role in lipid synthesis or breakdown in healthy adipose tissue and under disease conditions, including obesity, fatty liver disease, metabolic syndrome, and cancer formation/progression. STAT1/3/5 protein loss or hyperactivation, or differences in their activities within cell organelles due to expression and post-translational modifications, cause imbalances in metabolic regulation. NASH, NAFLD, and metabolic syndrome are closely associated with obesity, which predisposes to chronic inflammation associated with different, activated immune cell type infiltrations affecting many different cancer types, playing also a role in fuelling cancer via fat particle uptake. LDs emerge as critical organelles in this context, linking lipid storage to signalling, redox control, and cell death pathways such as ferroptosis and apoptosis. LDs play essential roles in cancer cells and in the TME/immunity, where the regulation of their formation and metabolism may be largely under mitochondrial or transcriptional control of STAT1/3/5, warranting further investigation. Thus, a better understanding of the fine-tuning of STAT1/3/5 molecules in lipid metabolism and knowledge of whether one could, e.g., interfere with their transport to mitochondria or LDs, or block, e.g., STAT phosphorylation to alter their function will be important for further work. In addition, future analysis should evaluate whether LDs can function as a STAT family reservoir that influences their intracellular organelle expression and activity, or whether this formation is more indirectly controlled by the transcriptional role of STATs. Overall, we lack detailed STAT profiles and activity insights within cell organelles, such as mitochondria, ER-GOLGI, peroxisomes, and the nucleus. We need to analyze different tissues and cell types, such as major muscles in the body, which are poorly investigated in the health or disease context, and to understand their key interactions, (neuro-)endocrine wiring, and how we might pharmacologically interfere with them. Here, obesity and T2D will complicate the analysis as outlined in this review on hepatic consequences of a fatty liver association in Insulin- or GH-resistant individuals that can progress to metabolic liver cancer or other associated comorbidities. Moreover, STAT1/3/5 proteins can promote anabolism or catabolism, independent of their role as transcription factors, and require tyrosine phosphorylation. In this context, de novo lipid synthesis, fatty acid β-oxidation, amino acid metabolism, glycolysis, the TCA cycle, energy generation, and building block synthesis for epigenetic reactions with the provision of methyl or acetyl groups are linked. The regulation of epigenetic chromatin function and the provision of energy groups such as ATP, GTP, and NADPH are linked to STAT protein function, localization, and organelle activity. A mechanistic understanding of these processes could lead to new therapeutic intervention strategies to disrupt or boost the organelle function of certain STAT family members. This might interfere with too much lipid depot accumulation or lipolysis. At the end, glucose, nucleotide, amino acid, and lipid metabolism need to be viewed in context, and the network of metabolic wiring with key proteins, including classic transcription factors, is a complicated, cell-type-specific matter.

The TCA cycle reflects high cellular respiration, but the HTC or the Warburg effect, as well as LDs generation, are all versatile “escape routes” for exhausted immune or cancer cells under selection pressure. Metabolism dictates whether they enter dormancy or senescence, providing slower forms of cellular metabolism. Ultimately, different forms of energy are required to drive growth, division, and the survival of cell types. Both cancer and immune cells need to be highly flexible, adapting to different harsh conditions. Future work should zoom in on STAT molecules and their pharmacologic blockade, stalling transport to mitochondria or LDs while sparing immune cell function or boosting immunity. To measure energy and major lipid classes, key metabolites or LDs with metabolic flux could unravel why LD generation or HTC is in Tango with STAT1/3/5 signalling. New models and assays are needed to identify more specific inhibitors that selectively block unwanted fat metabolism in diseased cells.

## Data Availability

No new data were created or analyzed in this study. Data sharing is not applicable to this article.

## Abbreviations

**ATGL**: Adipose Triglyceride Lipase; **PLIN**: Perilipin; **HSL**: Hormone Sensitive Lipase; **PKA**: cAMP-dependent Protein Kinase A; **MGL**: Monoacylglycerol Lipase; **CGI-58**: Comparative Gene Identification-58; **GR**: Glucocorticoid Receptor; **GHR**: Growth Hormone Receptor; **GH**: Growth Hormone; **JAK1/2**: Janus tyrosine kinases 1 and 2; **IL-6**: Interleukin-6; **IL-6R**: Interleukin-6 Receptor; **STAT1/3/5**: Signal Transducer and Activator of Transcription 1/3/5; **CD36**: Cluster of Differentiation 36 = multifunctional glycoprotein for lipid particle or FA uptake and binds also to many matrix proteins; **SR-B1**: Scavenger Receptor Class B Member 1; **FABPs**: Fatty Acid Binding Proteins; **FOXA2**: Hepatocyte Nuclear Factor 3-Beta; **FFA**: Free Fatty Acids; **IGF-1/2**: Insulin-like Growth Factor-1/2; **IR**: Insulin Receptor; **IGF-1/2**: Insulin-like Growth Factor-1/2; **LR**: Leptin Receptor; **TG**: Triglyceride; **TCA**: Krebs cycle; **DG**: Diacylglycerol; **MG**: Monoacylglycerol; **UPCs**: Uncoupling Proteins; **NASH**: Non Alcoholic Steatohepatitis; **NAFLD**: Non Alcoholic Fatty Liver Disease; **HCC**: Hepatocellular Carcinoma; **ChoCa**: Cholangiocarcinoma.
